# Supplementary material for: Hierarchical latent class models for mortality surveillance using partially verified verbal autopsies
Source: J R Stat Soc Ser A Stat Soc. 2025 Oct 21;189(3):1838–55. doi: 10.1093/jrsssa/qnaf164 (PMC13366219; doi:10.1093/jrsssa/qnaf164)
Supplement: qnaf164_Supplementary_Data [file qnaf164_supplementary_data.zip › qnaf164_Supplementary_Data/Zhu_JRSSA_Supplement.pdf]

# Supplementary Materials for "Hierarchical Latent Class Models for Mortality Surveillance Using Partially Verified Verbal Autopsies"

## Contents

**1 Additional summaries for the Brazil COVID-19 dataset 2**
1.1 Proportion of deaths with known causes in the resampled Brazil . . . . . . . . . . . . 2
1.2 Bootstrap uncertainty for the Matthews correlation coefficient (MCC) . . . . . . . . . 2
1.3 Sub-population sample size for the Brazil dataset . . . . . . . . . . . . . . . . . . . . . . 2

**2 Additional results for the analysis in the main paper 8**
2.1 Mean squared error of CSMF estimation . . . . . . . . . . . . . . . . . . . . . . . . . . . 8
2.2 Coverage probability . . . . . . . . . . . . . . . . . . . . . . . . . . . . . . . . . . . . . 9
2.3 Sensitivity to prior specification . . . . . . . . . . . . . . . . . . . . . . . . . . . . . . 10
2.4 Sensitivity to the number of latent classes . . . . . . . . . . . . . . . . . . . . . . . . . 10

# 1 Additional summaries for the Brazil COVID-19 dataset

## 1.1 Proportion of deaths with known causes in the resampled Brazil

Figure 1 shows the proportion of deaths with verified causes by age group and month in our analysis of the Brazil COVID-19 dataset.

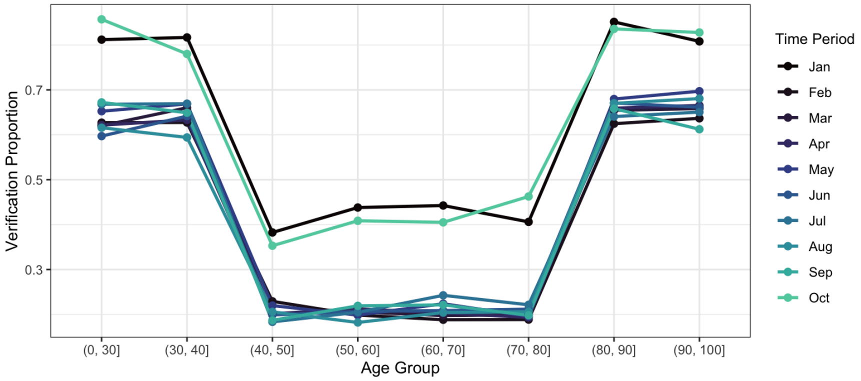


Figure 1: The verification proportion plot for each time/age sub-population under the verification mechanism in the numerical experiment.

## 1.2 Bootstrap uncertainty for the Matthews correlation coefficient (MCC)

Figures 2 to5 show Matthews correlation coefficient (MCC) of a subset of symptoms among deaths related to COVID-19 among deaths over all age and time groups, with point estimates and 90% bootstrap intervals for 1000 bootstrap samples of the Brazil VA dataset. Each plot shows the correlation coefficient between one specific symptom against four other symptoms. We only show the 16 pairs of symptoms as illustration due to space limit. Point estimates of the same 16 symptom pairs are presented in the main paper for a subset of age groups and time intervals. Missing data are removed in calculating correlation coefficient.

## 1.3 Sub-population sample size for the Brazil dataset

Figure 6 shows the sample size of the Brazil COVID-19 dataset by age group, sex, and month.

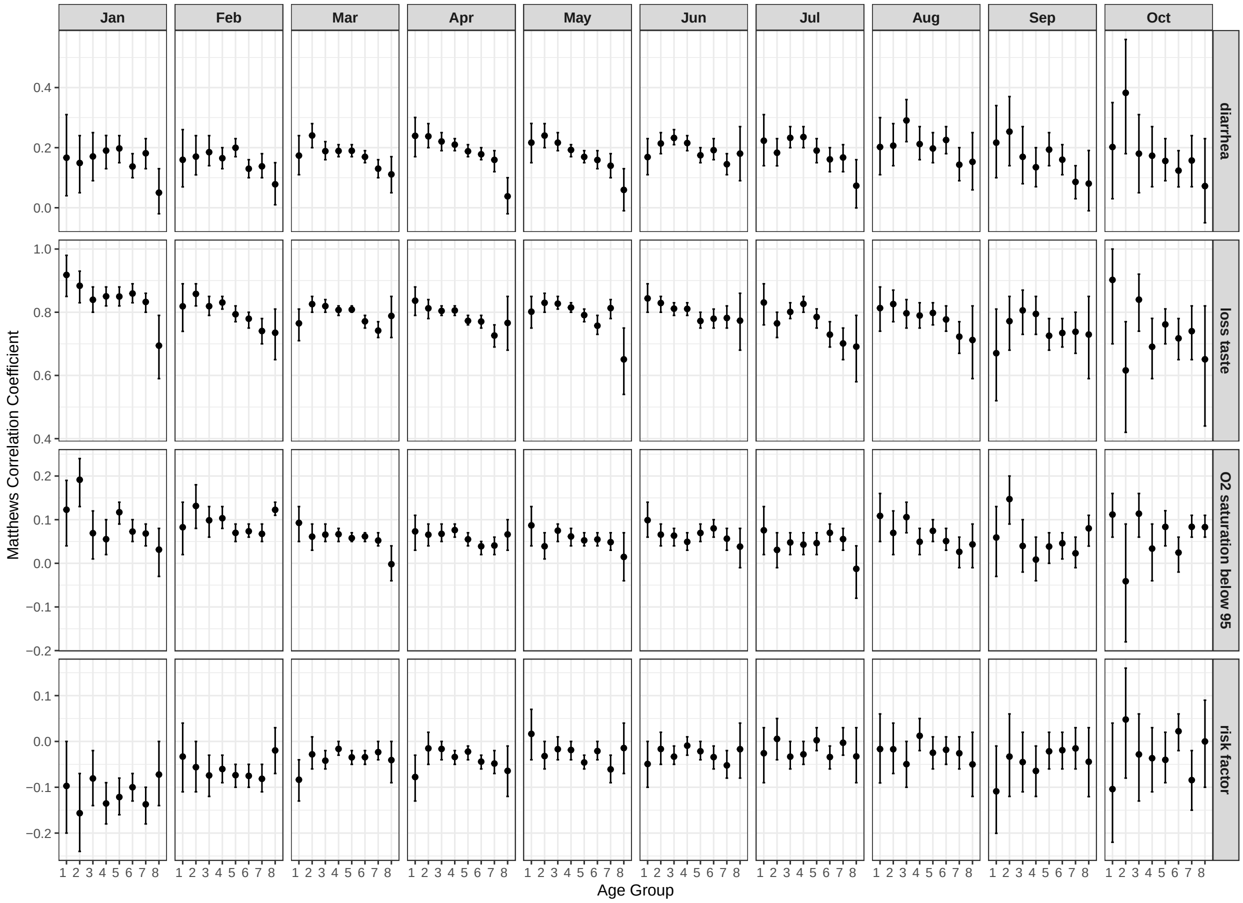


Figure 2: Bootstrap estimates of the Matthews correlation coefficient (MCC) and the associated 90% bootstrap intervals for 1000 bootstrap samples) for *loss smell* and four selected symptoms across sub-populations.

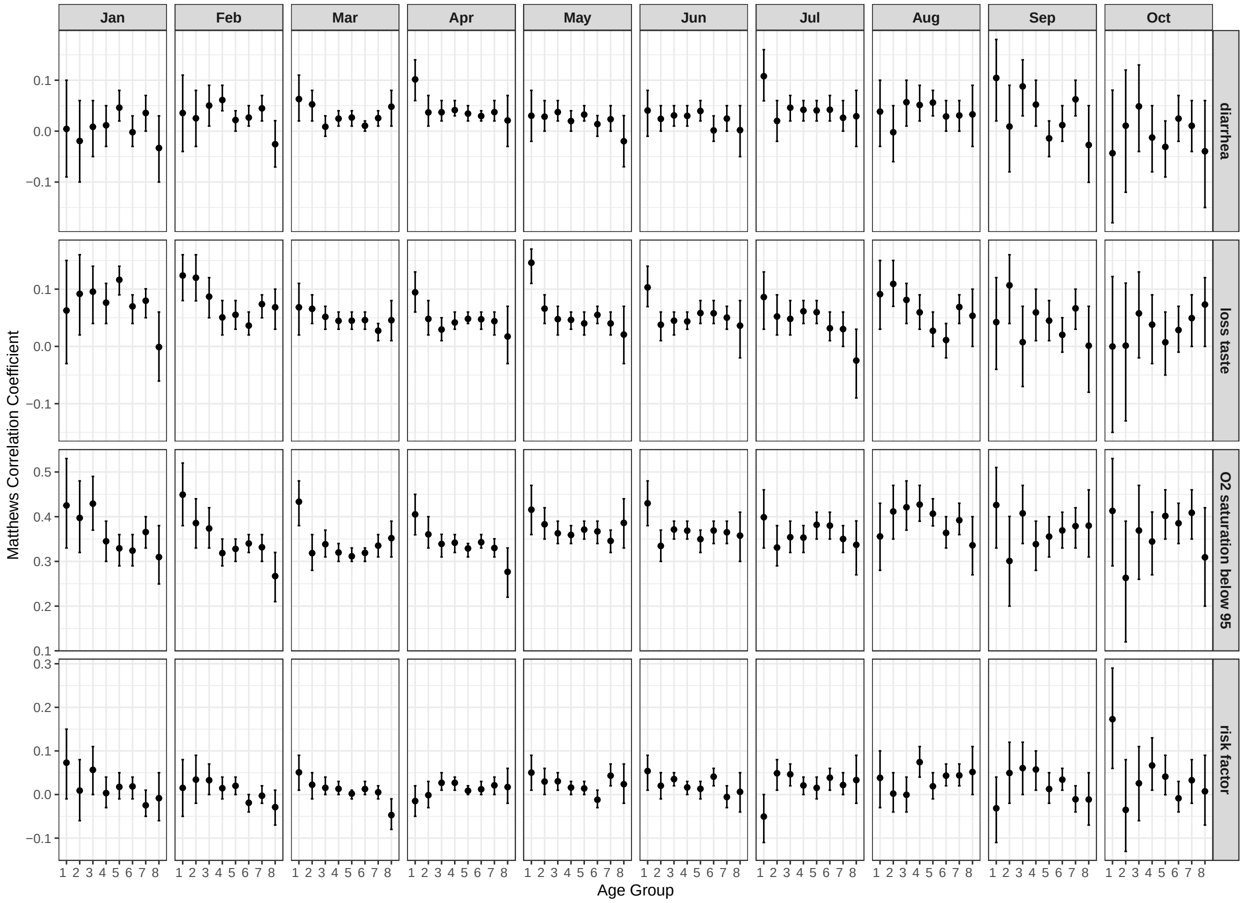


Figure 3: Bootstrap estimates of the Matthews correlation coefficient (MCC) and the associated 90% bootstrap intervals for 1000 bootstrap samples) for *dyspnea* and its selected symptoms across sub-populations.

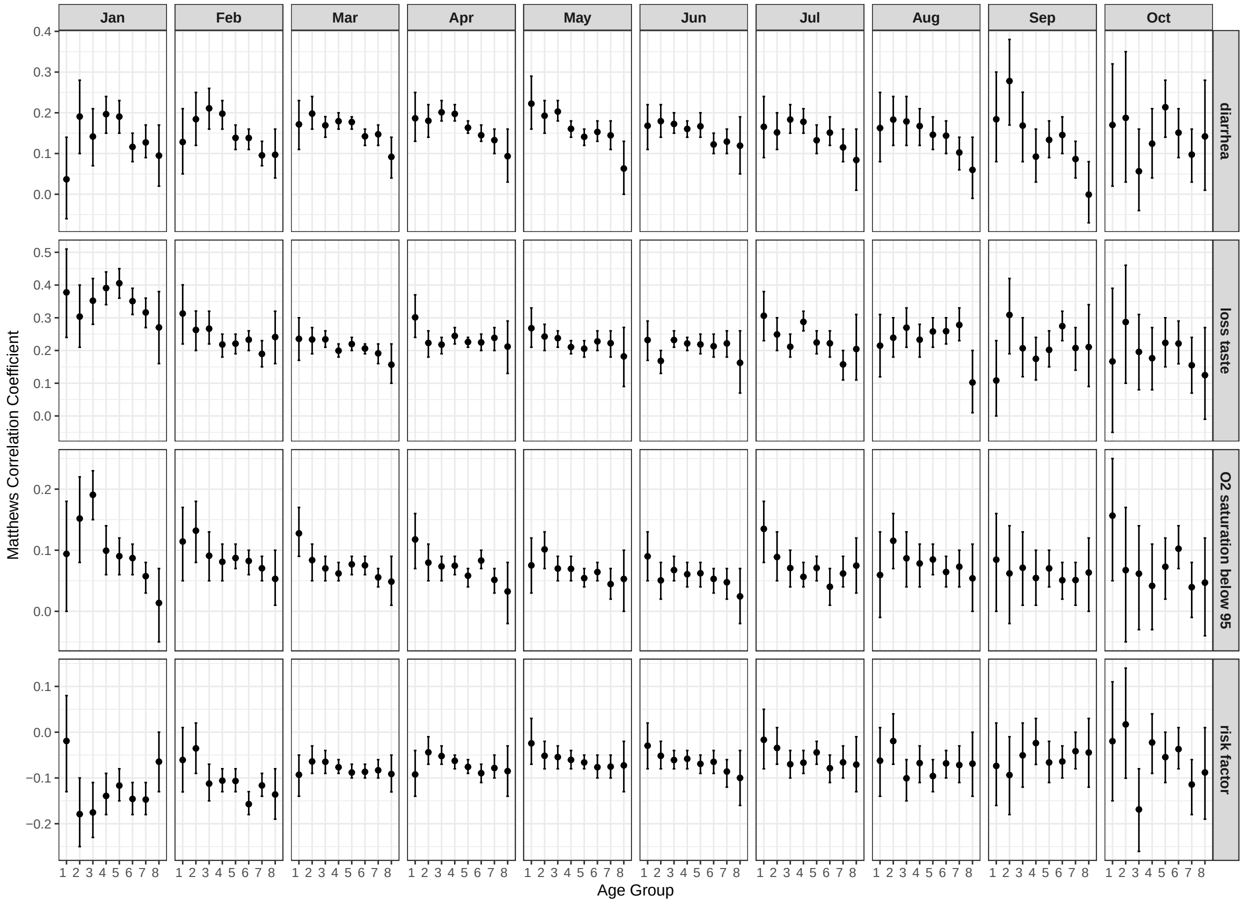


Figure 4: Bootstrap estimates of the Matthews correlation coefficient (MCC) and the associated 90% bootstrap intervals for 1000 bootstrap samples) for *sore throat* and its selected symptoms across sub-populations.

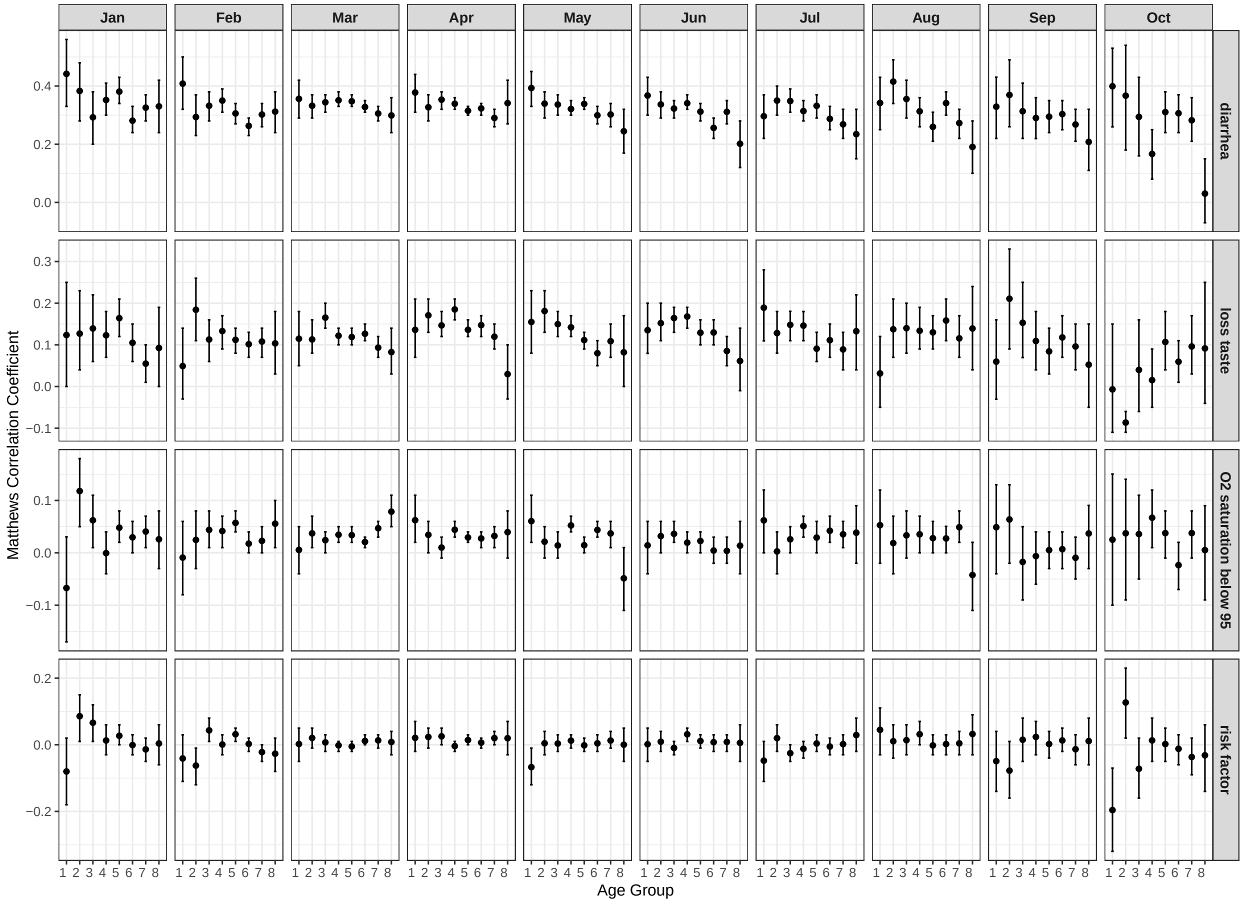


Figure 5: Bootstrap estimates of the Matthews correlation coefficient (MCC) and the associated 90% bootstrap intervals for 1000 bootstrap samples) for *vomiting* and its selected symptoms across sub-populations.

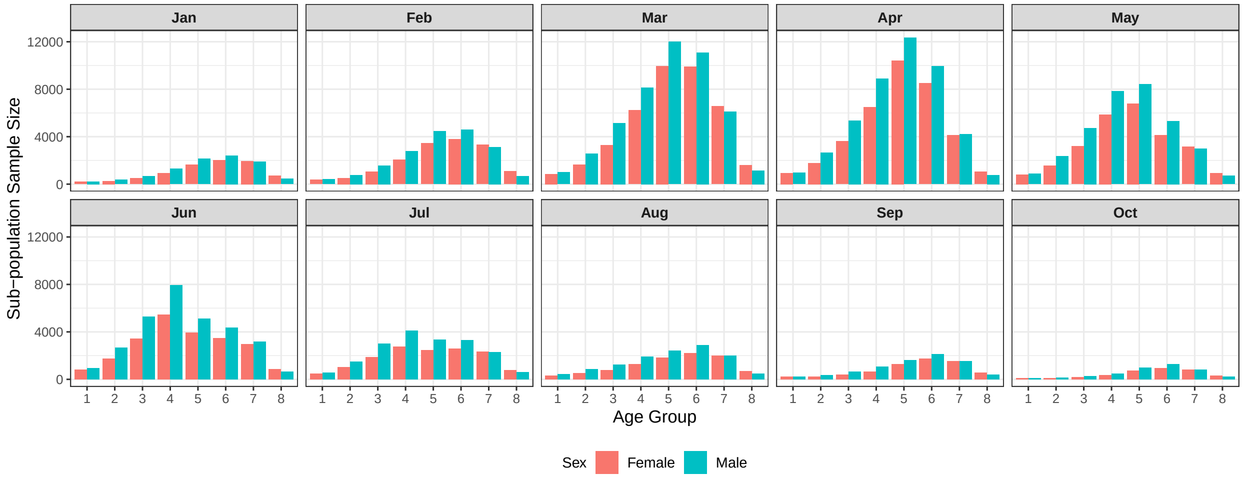


Figure 6: Sub-population sample size for the Brazil COVID-19 surveillance dataset by age group, sex, and month.

## 2 Additional results for the analysis in the main paper

### 2.1 Mean squared error of CSMF estimation

We also evaluate mean squared error (MSE) of the estimated subpopulation CSMFs over the 100 synthetic datasets. For each synthetic dataset, we compute

$$MSE = \frac{1}{STA}\sum_{s=1}^{S}\sum_{t=1}^{T}\sum_{a=1}^{A}(\hat{\pi}_{sta} - \pi_{sta})^2$$

for each model. Figure 7 shows the boxplot of the MSEs over the 100 datasets for each model. The unstratified model leads to significantly larger MSE than the other four stratified models, which are shown again in the right panel of Figure 7. Overall, estimates from the RW1 model show the smallest MSE.

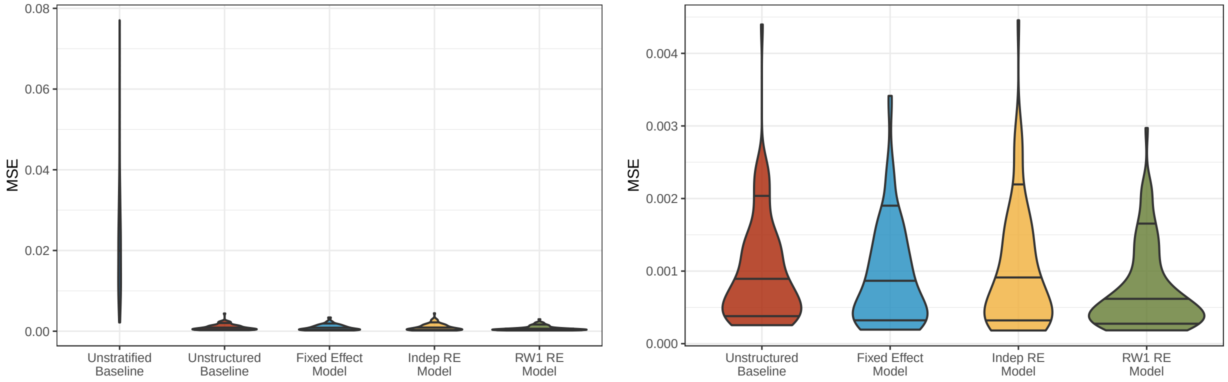


Figure 7: Mean squared error (MSE) of subpopulation CSMF estimates over 100 synthetic datasets resampled from the COVID-19 surveillance data from Brazil. Left: all five models considered in the paper. Right: excluding the unstratified baseline (the worst model in the left panel).

## 2.2 Coverage probability

Figure 8 shows the proportion of times when the 95% credible interval covers the true prevalence across the 100 synthetic datasets, for each month, age group, and sex. The four models considered in the paper yield similar average coverage probability across all subpopulations with 0.86 for the unstructured baseline model, 0.85 for the fixed effect model, 0.84 for the independent random effect model, and 0.87 for the RW1 model. The coverage are slightly lower than the nominal level, as the cause of death need to be estimated.

Across different subpopulations, we observe higher coverage in subpopulations where the verification probabilities are higher (first and last month, and first and last two age groups). Undercoverage of the subpopulations where the majority of deaths have unknown causes is also as expected. As can be observed in Figure 1, the sub-populations with low coverage are those with only around 20% of death having verified causes. In general, for smoothing models, even when we have a random sample of deaths in each subpopulation, the coverage rate for any given subpopulation will not be at the nominal level due to the bias introduced from shrinkage. This is well studied in the small area estimation literature (Burris and Hoff, 2020).

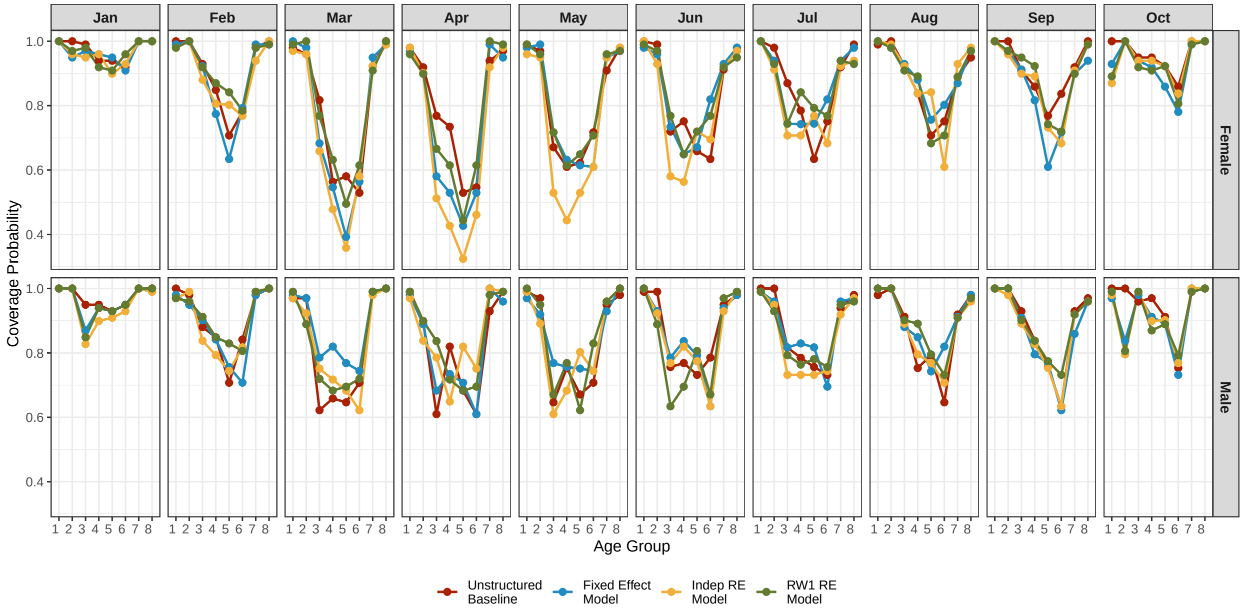


Figure 8: The coverage probability for sub-populations of stratification over sex, age, and time with 100 synthetic datasets resampled from the COVID-19 surveillance data from Brazil, under the four stratified models. The coverage probabilities for each sub-population vary across a wide range. However, the pattern aligns well with the verification mechanism shown in Figure 1: subpopulations with higher verification probabilities tend to have higher coverage, while those with lower verification probabilities tend to have lower coverage.

## 2.3 Sensitivity to prior specification

Figure 9 shows the posterior mean and 95% credible intervals of the estimated CSMF in one simulated dataset for different age groups, sex, and month under different priors for $\sigma^2$ under the RW1 model. We consider inv-Gamma priors that induce a 95% of prior mass on $(1/R, R)$ for the residual odds ratio with different choices of $R$. The results demonstrate that model performance is robust to the choice of $R$.

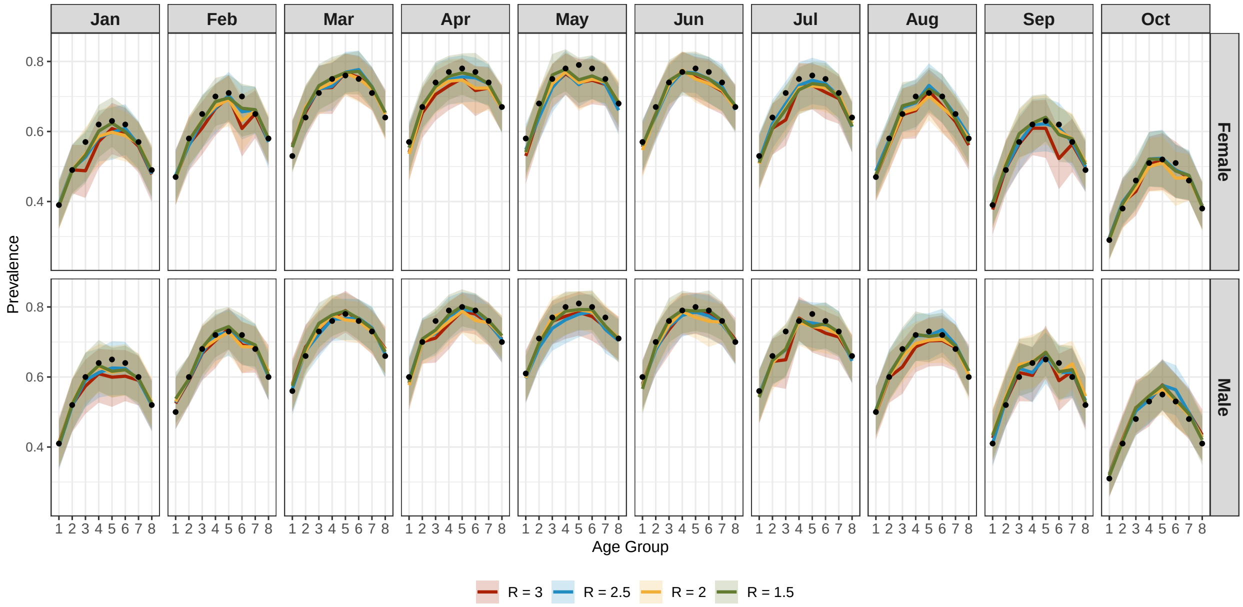


Figure 9: Prior sensitivity analysis of the RW1 model under different $R$ values, where $R$ defines the 95% prior mass for the residual odds ratio within $(1/R, R)$. $R = 2$ is used in the main paper.

## 2.4 Sensitivity to the number of latent classes

Figures 10 to 12 show the posterior mean and 95% credible intervals of the estimated CSMF using the RW1, independent random effect and fixed effect models in one synthetic dataset resampled from the COVID-19 surveillance data from Brazil for different age groups, sex, and month under $K = 5$, $K = 10$, $K = 15$, and $K = 20$. The results indicate that all the model fits are not sensitive to the choice of $K$ beyond $K = 10$, and the performance are stable with respect to latent class specification.

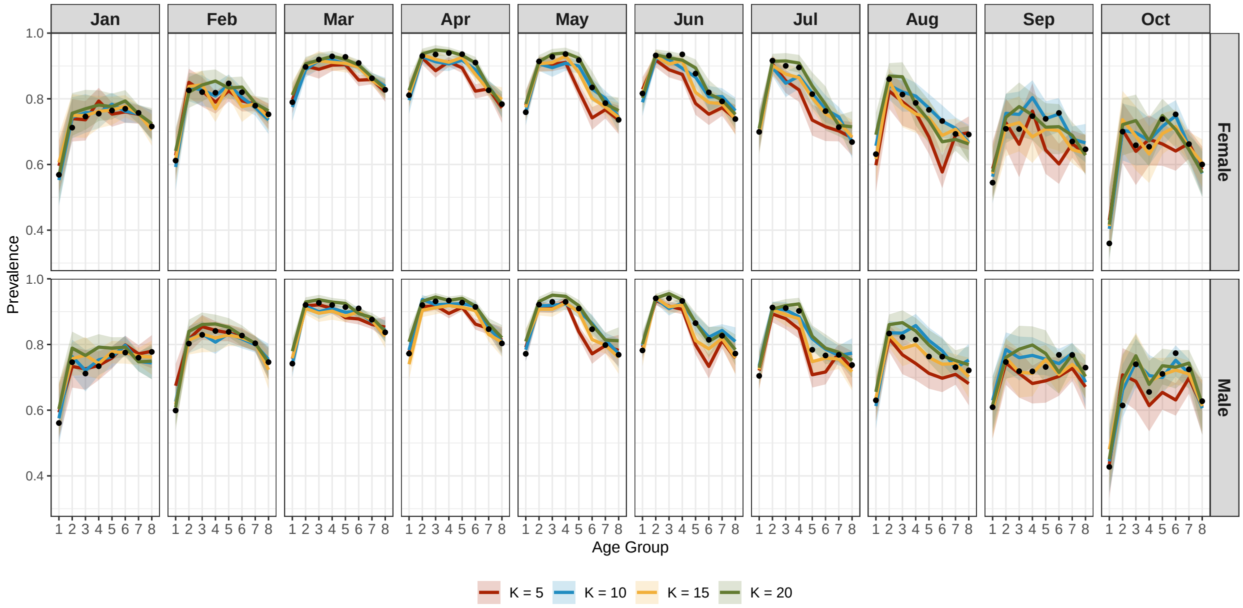


Figure 10: Sensitivity analysis of the *RW1* model under different choices of latent class number $K$ with the posterior mean and 95% credible intervals of the estimated CSMF for different age group, sex, and months, based on one synthetic dataset resampled from the COVID-19 surveillance data from Brazil. The true CSMFs are indicated by the black dots.

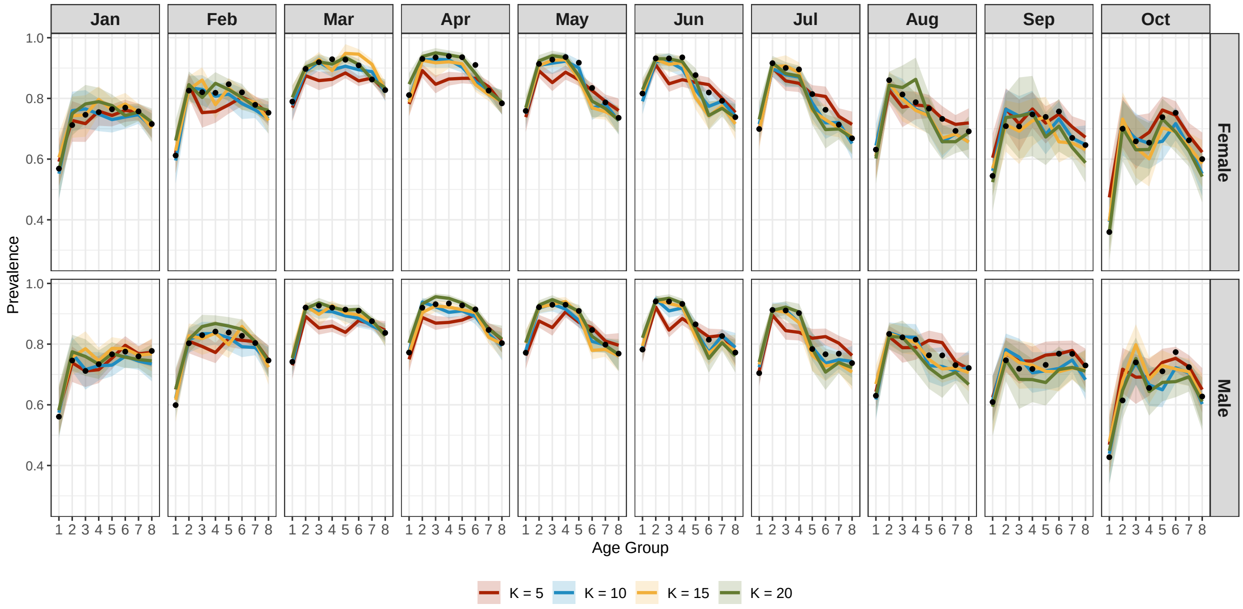


Figure 11: Sensitivity analysis of the *independent random effect* model under different choices of latent class number $K$ with the posterior mean and 95% credible intervals of the estimated CSMF for different age group, sex, and months, based on one synthetic dataset resampled from the COVID-19 surveillance data from Brazil. The true CSMFs are indicated by the black dots.

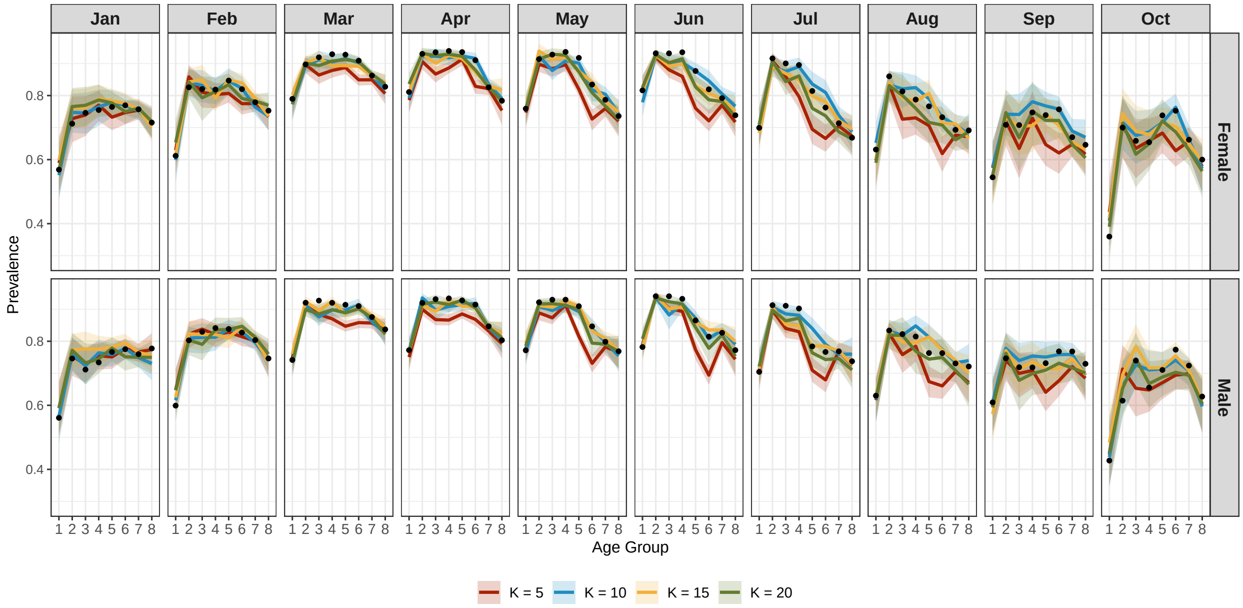


Figure 12: Sensitivity analysis of the *fixed effect* model under different choices of latent class number $K$ with the posterior mean and 95% credible intervals of the estimated CSMF for different age group, sex, and months, based on one synthetic dataset resampled from the COVID-19 surveillance data from Brazil. The true CSMFs are indicated by the black dots.

## References

Burris, K. C. and Hoff, P. D. (2020). Exact adaptive confidence intervals for small areas. *Journal of Survey Statistics and Methodology*, 8(2):206–230.
