# Supplementary material for: Hierarchical latent class models for mortality surveillance using partially verified verbal autopsies
Source: J R Stat Soc Ser A Stat Soc. 2025 Oct 21;189(3):1838–55. doi: 10.1093/jrsssa/qnaf164 (PMC13366219; doi:10.1093/jrsssa/qnaf164)

Examples of Hierarchical Latent Class Models for Mortality Surveillance Using Partially Verified Verbal Autopsies

Yu (Zoey) Zhu

2025-01-13

```r
# Import packages
library(Rcpp)
library(MCMCpack)
```

```
## Loading required package: coda

## Loading required package: MASS

## ##
## ## Markov Chain Monte Carlo Package (MCMCpack)

## ## Copyright (C) 2003-2025 Andrew D. Martin, Kevin M. Quinn, and Jong Hee Park

## ##
## ## Support provided by the U.S. National Science Foundation

## ## (Grants SES-0350646 and SES-0350613)
## ##
```

```r
library(ggplot2)
library(MetBrewer)
library(plyr)
library(MASS)
library(mnormt)
library(BayesLogit)
library(gsignal)
```

```
##
## Attaching package: 'gsignal'

## The following objects are masked from 'package:stats':
##
##     filter, gaussian, poly
```

```r
library(gtools)
```

```
##
## Attaching package: 'gtools'

## The following objects are masked from 'package:MCMCpack':
##
##     ddirichlet, rdirichlet
```

```r
library(tidyr)
library(caret)
```

```
## Loading required package: lattice
```

Simulation without aggregation example

In this report we show one simple example of simulation study without aggregations. The processes are (1) loading the model fitting functions and simulated data generating function. (2) setting up the model parameters and verification mechanism argument. (3) generating true prevalence and simulate the data. (4) fitting the models. (5) visualize the results, i.e, posterior mean and CI for the models comparing with the simulated true prevalence. The simulation study with aggregations to the time and population levels examples are attached in `analysis_no_aggregate_sim_data.R`.

Import functions:

```
## Rcpp dependencies
sourceCpp("../model/Rcpp/fit_model_BL.cpp")
sourceCpp("../model/Rcpp/fit_model_with_structured_prior.cpp")
## Models
source("../model/fit_model_BL.R")
source("../model/fit_model_with_structured_prior.R")
## Data generation function
source("../simulation/sim_with_sex_time_age.R")
```

Set up the model parameters and verification mechanism argument:

```
# Set up
n_Sex = 2
n_Age = 8
n_Time = 10

n_subsize = 100 # sample size of each sub-population
q = 10
K = 10

DEPEND_ON_Y = FALSE # Case (i)
```

Generate pi_true as prevalence:

```
p_S = c(0.2, 0.3)

x <- 1:n_Time
p_T <- (-6 + 10*x - x^2) / n_Time / 2

x <- 1:n_Age
p_A = (-6 + 10*x - x^2) / n_Age / 2

Nsta = n_Sex * n_Time * n_Age
pi_true = rep(NA, Nsta)
S_ind = c(rep(0, n_Time * n_Age), rep(1, n_Time*n_Age))
T_ind =  rep(rep(1:n_Time, each = n_Age), n_Sex)
A_ind =  rep(rep(1:n_Age,  n_Time), n_Sex)
for(i in 1:Nsta){
  pi_true[i] = round(expit(-1 + p_S[S_ind[i]+1] + p_T[T_ind[i]] + p_A[A_ind[i]]), 2)
}
```

Fit the models:

```
# generate simulated dataset
sim_data = sim_with_sex_time_age(pi_true, n_subsize = n_subsize, q = q, n_Sex = n_Sex, n_Time = n_Time,
```

```
E = 2000
BURN_IN = 1000
# fit models
fitted_model_BL = fit_model_BL(sim_data, E = E, BURN_IN = BURN_IN)
```

```
## Loading required package: RcppArmadillo
```

```
## ....................
```

```
fitted_model_Fixed = fit_model_with_structured_prior(sim_data, K = K, E = E, BURN_IN = BURN_IN, STRUCTU
```

```
##
## Attaching package: 'pracma'
```

```
## The following object is masked _by_ '.GlobalEnv':
##
##     logit
```

```
## The following object is masked from 'package:gtools':
##
##     logit
```

```
## The following objects are masked from 'package:gsignal':
##
##     conv, detrend, fftshift, findpeaks, ifft, ifftshift
```

```
## The following object is masked from 'package:MCMCpack':
##
##     procrustes
```

```
## ....................
```

```
fitted_model_Indep = fit_model_with_structured_prior(sim_data, K = K, E = E, BURN_IN = BURN_IN, STRUCTU
```

```
## ....................
```

```
fitted_model_RW = fit_model_with_structured_prior(sim_data, K = K, E = E, BURN_IN = BURN_IN, STRUCTURED
```

```
## ....................
```

Visualize the posterior mean and CI of prevalence for the models:

```
est0 <- data.frame(sex = c(rep("F", n_Time * n_Age), rep("M", n_Time * n_Age)),
                   time = rep(rep(1:n_Time, each = n_Age), 2),
                   age = rep(rep(1:n_Age, n_Time), 2),
                   prev = fitted_model_BL$bias+sim_data$param$pi,
                   lower = apply(fitted_model_BL$poster_sample$sample_pis,2, function(x) quantile(x, 0
                   upper = apply(fitted_model_BL$poster_sample$sample_pis,2, function(x) quantile(x, 0
                   type = "BL")
est1 <- data.frame(sex = c(rep("F", n_Time * n_Age), rep("M", n_Time * n_Age)),
                   time = rep(rep(1:n_Time, each = n_Age), 2),
                   age = rep(rep(1:n_Age, n_Time), 2),
                   prev = fitted_model_Fixed$bias+sim_data$param$pi,
                   lower = apply(fitted_model_Fixed$poster_sample$sample_pis,2, function(x) quantile(x
                   upper = apply(fitted_model_Fixed$poster_sample$sample_pis,2, function(x) quantile(x
                   type = "Fixed")
est2 <-  data.frame(sex = c(rep("F", n_Time * n_Age), rep("M", n_Time * n_Age)),
                    time = rep(rep(1:n_Time, each = n_Age), 2),
                    age = rep(rep(1:n_Age, n_Time), 2),
                    prev = fitted_model_Indep$bias+sim_data$param$pi,
```

```
                      lower = apply(fitted_model_Indep$poster_sample$sample_pis,2, function(x) quantile
                      upper = apply(fitted_model_Indep$poster_sample$sample_pis,2, function(x) quantile
                      type = "Indep")
est3 <-  data.frame(sex = c(rep("F", n_Time * n_Age), rep("M", n_Time * n_Age)),
                      time = rep(rep(1:n_Time, each = n_Age), 2),
                      age = rep(rep(1:n_Age, n_Time), 2),
                      prev = fitted_model_RW$bias+sim_data$param$pi,
                      lower = apply(fitted_model_RW$poster_sample$sample_pis,2, function(x) quantile(x,
                      upper = apply(fitted_model_RW$poster_sample$sample_pis,2, function(x) quantile(x,
                      type = "RW")


param <- data.frame(sex = c(rep("F", n_Time * n_Age), rep("M", n_Time * n_Age)),
                      time = rep(rep(1:n_Time, each = n_Age), 2),
                      age = rep(rep(1:n_Age, n_Time), 2),
                      prev = sim_data$param$pi,
                      lower = NA,
                      upper = NA)

emp.obs <- aggregate(sim_data$data$Y.t ~sim_data$data$A + sim_data$data$Ti + sim_data$data$S  , FUN = me

observed <- data.frame(sex = c(rep("F", n_Time * n_Age), rep("M", n_Time * n_Age)),
                         time = rep(rep(1:n_Time, each = n_Age), 2),
                         age = rep(rep(1:n_Age, n_Time), 2),
                         prev = emp.obs$`sim_data$data$Y.t`,
                         lower = NA,
                         upper = NA)


out <- rbind(est0, est1, est2, est3)

  time_index = month.abb[1:10]
  out$time <- time_index[out$time]
  out$time <- factor(out$time, levels = time_index)
  param$time <- time_index[param$time]
  param$time <- factor(param$time, levels = time_index)

  observed$time <- factor(observed$time, levels = 1:n_Time)
  out$age <- factor(out$age, levels = 1:n_Age)
  param$age <- factor(param$age, levels = 1:n_Age)
  observed$age <- factor(observed$age, levels = 1:n_Age)
  out$sex <- factor(out$sex, levels = c("F", "M"))
  param$sex <- factor(param$sex, levels = c("F", "M"))
  observed$sex <- factor(observed$sex,  levels = c("F", "M"))
  out$type <- factor(out$type, levels = c("BL", "Fixed", "Indep","RW"))


  out$type <- revalue(out$type, c("BL" = "Unstructured",
                                  "Fixed" = "Fixed Effect",
                                  "Indep" = "Indep RE",
                                  "RW" = "RW1 RE"))
  out$sex <- revalue(out$sex, c("M" = "Male",
                                "F" = "Female"))
```

```r
  param$sex <- revalue(param$sex, c("M" = "Male",
                                    "F" = "Female"))

  cols <- met.brewer("Juarez", n = 6, type = "discrete")[-4]


g_posterior <- ggplot(subset(out, type %in% c("Unstructured", "RW1 RE")), aes(x = age, y = prev))  +
    geom_ribbon(aes(ymin = lower, ymax = upper, fill = type, group = interaction(time, type)), alpha =
    geom_line(aes(color = type, group = interaction(time, type)), linewidth = 1.05) +
    geom_point(data = param, aes(x = age, y = prev), col = "black", size = 1.2) +
    facet_grid(sex ~ time) +
    scale_color_manual("Model", values = c("#d95f02", "#1b9e77"))+
    scale_fill_manual("Model", values = c("#d95f02", "#1b9e77"))+
    theme_bw() +
    xlab("Age Group") + ylab("Prevalence") +
    theme(axis.text.x = element_text(size = 10), # Make X axis labels (age) bold
          strip.text = element_text(face = "bold",size = 10),  # Make facet labels (time) bold
          legend.text = element_text(size = 10))+
    theme(legend.position = "bottom", strip.text = element_text(size = 12))

print(g_posterior)
```

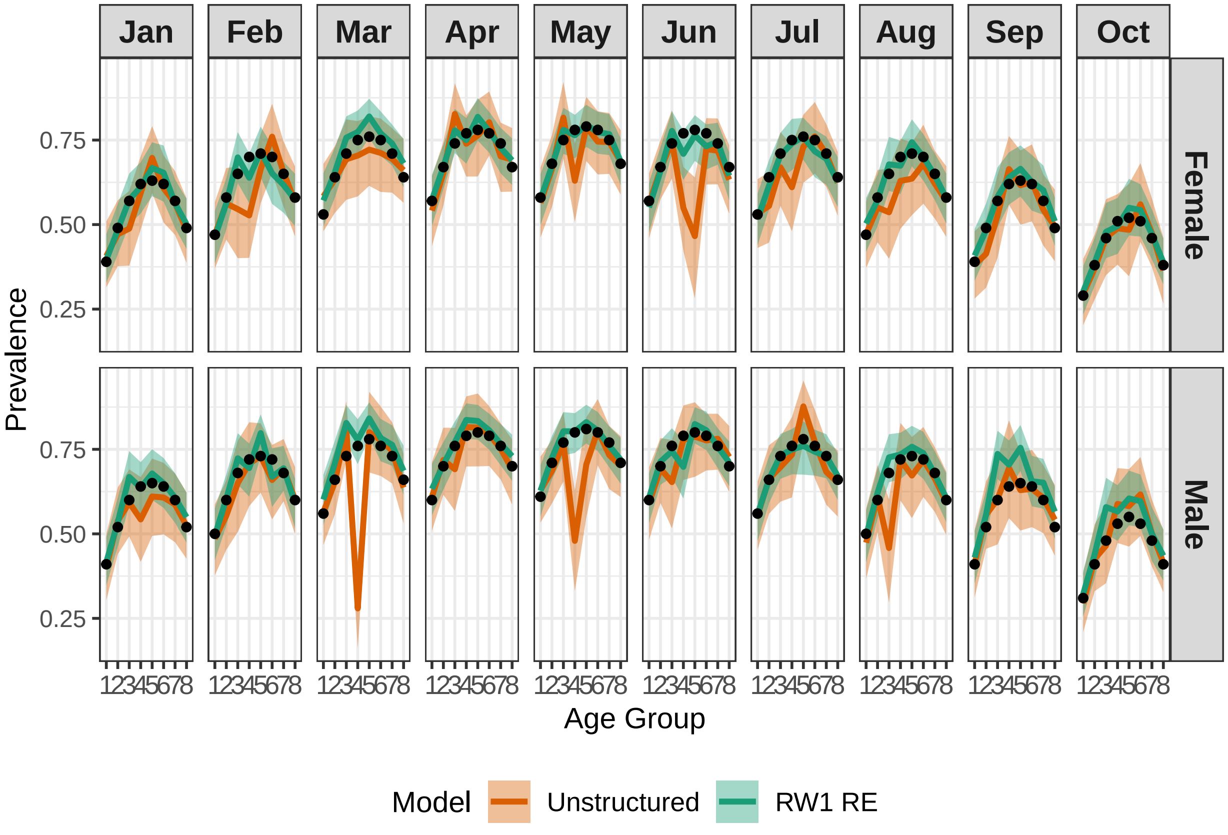

Supplement: qnaf164_Supplementary_Data [file qnaf164_supplementary_data.zip › qnaf164_Supplementary_Data/COVID_VA_codes/reproducible_examples/examples.pdf]
